# Psychological Effects of COVID-19 and Its Measures in Nepalese Medical Students

**DOI:** 10.31729/jnma.5276

**Published:** 2020-10-31

**Authors:** Anima Bhandari, Nisha Bhatta

**Affiliations:** 1 Kathmandu University School of Medical Sciences, Dhulikhel, Nepal

**Keywords:** *psychological effects*, *medical student*, *COVID-19*

## Abstract

Having to listen to the devastating daily news brought by COVID-19 since the first case was reported on 23rd January 2020 in Nepal, it has pushed the country into various crises. The issue of psychological health has been overlooked during this crisis.COVID-19 has sabotaged the psychological health of general people and more importantly medical students. It has disrupted the academics and clinical rotations of medical education. The prospect of being the future health care personnel without adequate training has destroyed the confidence and aspiration andin addition, joining the frontline against this type of deadly virus with little preparedness has instilled fear and uncertainty among them. So, concerning the context, this article focuses on the psychological effects faced by medical students and some ways to overcome this issue.

## INTRODUCTION

The novel coronavirus disease (COVID-19) was first detected in the country China (Wuhan, Hubei Province) in December 2019. From that moment, it began to spread first in China, and shortlyafterward throughout the world. Confirmed cases and deaths grew rapidly, and on August 11th there have been quite 19,936,210 confirmed cases worldwide and quite 732,499 people have died from it.^1^ This situation has produced a notable emotional impact on medical workers and students and thegeneral population, with important symptoms of anxiety, stress, and depression.^2^

The cases in Nepal are still following a step ladder pattern reporting 23,948 confirmed cases with 83 deaths till 11th August 2020.^3^ Because therate of spread is increasing day by day, developing nations like Nepal decided that lockdown is theonly option available to slacken the speed of spreading the infection, which was started on 24th March 2020. For many Nepalese, this is often the primaryexperience of an emergency with an indiscernible agent, resulting ingreat uncertainty and significant adverse consequences for psychological state.

Every sector of the country has been paralyzed parallel to the increasing number of cases where the casualty with no doubt is medical students as well. Medical education and clinical experiences being their prime responsibility, the pause of it brings potential risk to their psychological health.

## GLOBAL SCENARIO OF PSYCHOLOGICAL EFFECTS IN MEDICAL STUDENTS

Assessment of the psychological stateof medical students (n = 7143) from Changzhi school of medicine, China, demonstrated self-reported anxiety in 25% of participants.^4^ Another study on health care provider students (n = 1442) including medical students at Sichuan University, China, showed the presence of COVID-19-related psychological distress in 27% participants with 11% exhibiting an acute stress reaction to COVID-19.^4^ Another global study assessing the psychological stateof medical students from 12 different countries demonstrated alarming high rates of psychological state problems, burnout, drug abuse, and mental stress in medical students.^5^

## PSYCHOLOGICAL CHALLENGES FACED BY NEPALESE MEDICAL STUDENTS

Medical students entangled between the pandemic and the paralyzed education routine are experiencing increasing anxiety as COVID-19 gradually affects their mental, physical, and emotional well-being. Longstanding social distancing can have negative effects on their psychological state. A current pandemic can worsen already existing psychological stateconditions within them. The present scenario of the country along with the interrupted daily routines of the medical students, their psychological health forms a curve involving mood swings to the advanced level of depression.

Medical students already have stress due to the longer duration of course. Pause in it, pushes them to become more anxious.It is also documentedthat medical students experience stress especially before and throughthe examinations.^6^ The examinations were postponed due tothe lockdown and therefore theactual date of the exam is awaited. During thiscontext, many students might be undergoing mental stress and there's a robust got toconsider their psychologicalstatus.^7^

As medical students are retracted from clinical experiences, their entire curriculum is transitioned to virtually-delivered format leading to no on-campus activity, exams being offered online and licensure exams being delayed. Students who must continue patient contact through this pandemic are limited due to lack of adequate personal protective equipment (PPE) thus compromising their comprehensiveness in clinical experiences. Additionally, certain specialties require medical students to achieve letters of recommendation from non-home institutions for his or herresidency applications needing visiting electives. These electives are currently not feasible because of existing travel bans thereby impacting medical students applying for 2021 residency training.^8^

With the numbers of deaths increasing day by day and with the news and social media flooding with COVID-19 discussions, it's hard to remainunaffected. Adapting to what seems to be the new normal of medical education with a lack of on-campus learning, absence of peer interactions, the omission of direct patient care involvement increases stress among medical students. Medical students are a vulnerable group of people globally and according to one study are known to display higher rates of depression, suicidal ideation, and stigmatization around depression, anxiety, stress, and also areless likely to seek support.^8–9^

Nepal being a developing nation, does not have a stable electricity and internet facility. Unable to attend the online class which is being conducted by the universities gives stress to the medical students. Students with an economic crisis might feel issues, leading to depression. Summing up all these issues, suicidal attempts may arise within fragile minds who easily cannot accept the changes. It is therefore important to safeguard the psychological health of medical students with an effective plan to support their wellness and education.

## MEASURES TO OVERCOME THE PSYCHOLOGICAL EFFECTS

Due to the overwhelming impact of COVID-19 on the psychologicalstateof the medical students, simple to multidimensional and multilevel interventions could beneeded. Learning from China's experiences about COVID-19 related psychological statechallenges in medical students, medical schools globally should bolster psychological statesupport for his or hermedical students. It's a time for each individual, medical students, communities, and nation to think aboutdifferent strategies for a sound psychological state of medical students during these times of uncertainties brought by the pandemic.

1. **Policies Formulation:**
  - Immediate and future actions should be enlisted within theprotocol by the governmentto mitigate the psychological stateconsequences and similar future situations with better monitoring systems.^10^
  - Surveys should be conducted frequently by different institutions and associated bodies to figure out the prevalent problems and actions that should be taken to manage them accordingly.^10^
2. **Implementation of Policies:**
  - Arrangement of sound health facilities to the front liners, adequate PPEs distribution, and positive environment to the medical students with a motto reducing their fear of front liners being at uncontrolled risk.
  - Arrangement of yoga, breathing exercises, and recreational programs should be covered up.
  - Provision of data packages to the students where electricity and internet facilities are lagging behind.
3. **Creating awareness:**
  - Medical Students, as well as all the citizens of the nation, should be made aware of possible adverse effects of the pandemic and thelockdown and suggest everyone take possible measures at their stand.^10^
  - Time to time awareness of the medical students about the amplification of stress due to the continuous use of cell phones, desktops should be done. Conduction of creative programs with rewards, motivation on indoor games, and involvement in extracurricular activities should be emphasized.
  - Awareness about discouraging distressing footage and prevention of overexposure to anxietyprovoking media should be done.
  - Social sites, newspapers, and adequate media coverage on both rural and urban areas for awareness and motivation should be done.
  - Limiting multiple separate communications about COVID-19 response and having one single robust weekly communication sharing the action plan may be a great wayto decrease student anxiety and conveysome organizational order amid chaos.^8^
4. **Role of medical school:**
  - Medical schools should stillstay connected with students virtually. Checking in emails to stayan open communication may be a good way to permitstudents to reachout if needed. Weekly class meetings on a webplatform will allow students to interactwith one anotherand share thoughts and struggles, it will allow them to connect despite social distancing.
  - The students should be made sure that there willnot be any loss of the year.
  - Offering psychological state visits, preferably freed fromcharge, and with the powerof maintaining anonymity will encourage medical students to seek help and receive professional assistance to wade throughtheir negative emotions
  - Provision of the psychiatrist for the online counseling of both the medical students and the parents as possible. Motivation should be prioritized for positivity regarding the courses and the schedules.^7^
  - The university also should monitor the students even after the lockdown because ittakes time for them to normalize themselves after the long, unexpected break in their studies. Continuous monitoring, offering to counsel the needy students will help tokeep the studentsmentally healthy and have the bestin personal and business life.^7^

## WAY FORWARD

The impact on psychological health brought by the pandemic COVID-19 and ongoing nationwide lockdown is a major unvoiced issue. Considering this, the measures and protocol should be followed to adjust with the new normal. The difficult time demands the joint effect of every medical students, institutions and the nation to have a balanced psychological state.

## Conflict of Interest

**None.**
